# The Universal Triangle to Ensure Health Security

**DOI:** 10.3389/ijph.2022.1605443

**Published:** 2022-10-19

**Authors:** Saharnaz Sazgarnejad, Amirhossein Takian

**Affiliations:** ^1^ School of Public Health, Tehran University of Medical Sciences, Tehran, Iran; ^2^ School of Medicine, Tehran University of Medical Sciences, Tehran, Iran; ^3^ Department of Global Health and Public Policy, School of Public Health, Tehran University of Medical Sciences, Tehran, Iran; ^4^ Department of Health Management, Policy and Economics, School of Public Health, Tehran University of Medical Sciences, Tehran, Iran; ^5^ Health Equity Research Centre (HERC), Tehran University of Medical Sciences, Tehran, Iran

**Keywords:** COVID-19, health security, universal health coverage, global solidarity, sustainable development goals

Historically, health security has been one of the most critical aspects of human needs. Several important periods of human life have been threatened by infectious diseases epidemics and pandemics, e.g., imposing quarantine against the plague crisis in the 14th century, and the Ebola outbreak in West Africa in 2014 [1]. Since December 2019, the ongoing COVID-19 pandemic has led to over 617 million confirmed cases and 6.5 million recorded deaths globally [2]. Therefore, once again, the weaknesses and strengths of the global health structures in dealing with health crises have been revealed.

The COVID-19 pandemic has crystalized how far-reaching epidemics can impose financial and economic burdens on societies [3], while people in low and middle-income countries (LMICs) might become more vulnerable compared with residents in high-income countries (HICs) during crises [4]. Most countries, particularly high-income settings, had initially taken a more nationalistic than a global approach to combat the crisis, which led to failed attempts to achieve global solidarity and inadequate effective interventions to timely terminate the pandemic. The semantic differences in terms of health security across countries, especially in the realm of health-related policymaking, have compromised global success in dealing with crises. Patriotic actions and prioritizing nationalism over global solidarity by industrialized nations, mainly to protect their citizens, have compromised health security and global success against external threats such as bioterrorism and infectious epidemics. Let alone, many LMICs are still mostly concentrated on expanding healthcare services, rather than nurturing health into upstream policymaking, which is essential to deal with the complex threats to citizens’ well-being in the 21st century [5].

The Global Health Security Index of 2021 suggests that even countries with higher scores are not prepared enough to combat future pandemics [6]. The significant differences among countries especially in healthcare infrastructure, might lead to considerable variations in their response to crises. It is essential to strengthen the capacity of LMICs for meaningful national surveillance and improving international data-sharing [7]. As countries’ concerns vary regarding health security, the timely control of future crises might need a paradigm shift from health security at the country level to the “human beings’ health security” at the global scale.

Subsequently, defining clear, precise, and more practical definitions, as well as paying more attention to a high-risk population that experiences complex dimensions of insecurity, including health security, is essential to determining and implementing future policies. It is also crucial to navigate a paradigm shift from emphasizing the differences among countries to paying attention to similarities among all individuals. Respectively considering the multifaceted nature of health issues, acknowledging the common vulnerabilities of human beings in front of external threats, such as infectious epidemics, and social determinants of health could be considered a momentous measure to achieve global health security [8]. Such an approach renders a fundamental shift from labeling poor countries as culprit causes of global health insecurity, toward partnering with citizens in all settings, including the most vulnerable, for the implementation of policies in achieving global solidarity to fight against external threats.

Future pandemics are likely to happen even soon [9]. To determine the well-being and safety of all human beings, as global citizens, we advocate reevaluating the existing indices in examining the state of health security at global, national, and individual levels. Health is a basic human right and the pillar for sustainable societies. To achieve health equity and universal wellbeing regardless of borders and in accordance with sustainable development goals, we recommend: 1) Enhancing intersectoral and international collaboration to improve the risk perceptions and mutual responsibilities of governments and citizens, through fostering meaningful platforms for stakeholders’ engagement; 2) Empowering countries and international organizations in sharing information, technology, and health services toward developing health security indicators; and 3) Developing a series of risk-based approach indicators to evaluate and monitor the state of global health security with greater emphasis on vulnerable and at-risk populations. Universal health coverage (UHC) is still a very valid ultimate goal for all health systems during the post-COVID-19 era anywhere. We call for universal health preparedness and universal health solidarity to achieve UHC, the universal triangle, in rebuilding the resilient health systems of future.
